# An Account of the Good Effects of Calomel in a Case of Obstructed Menses

**Published:** 1786

**Authors:** James Watson

**Affiliations:** Surgeon of the Second Regiment of Dragoons, and Member of the Corporation of Surgeons of London


VI. *An Account of the good Effects of Calomel in a Cafe of obftructed Menfes. Communicated in a Letter to Dr.* Simmons *by Mr.* James Watfon, *Surgeon of the Second Regiment of Dragoons, and Member of the Corporation of Surgeons of London.*

IN the month of March laft I was defired to vifit Mrs. ——, aged twenty-three years, who gave me the following account of her fituation, viz. that, about twenty-feven months before, fhe had been feized with pains in her limbs, attended with fome degree of fever, and which happened at the time the menftrual difcharge fhould have come on. This fhe confidered as trivial, and had imputed it to a flight cold. The pains and fever foon left her; but from that period fhe had never menftruated, or indeed had had the leaft appearance of fuch a difcharge. Her health had been better and worfe, fhe faid, till within three months of my feeing feeing her, when ſhe complained of being ſo languid and feeble, that her uſual occupation ſhe was now unable to go about. Her appetite was gone; what aliment ſhe took was ſometimes brought up by vomiting, or paſſed through her in an undigeſted ſtate: her legs were become œdematous, and an ugly ſore had broke out on one of them, which diſcharged a filthy ſerum. She complained alſo of an unuſual weight and pain about the uterus. Her ſight was much affected, and her hearing not ſo good as when in health: ſhe had no deſire for food, or even to take drink, and only did ſo when urged to it.

As the loſs of tone (and, I would add, irritability) in the ſyſtem was evidently the remote cauſe of all her complaints, to reſtore that, and, by ſo doing, to excite the action of the uterine veſſels, and bring back the menſtrual diſcharge, ſeemed to be the indications of cure. Every thing of the tonic kind was tried, with the addition of full diet, &c. Emmenagogues were alſo had recourſe to, but without producing the leaſt favourable change. Purgatives were, in this caſe, (from the relaxed ſtate of the patient) I conceived, inadmiſſible; however, having in two two inſtances of ſuppreſſed menſes made uſe of calomel (though only as a purgative) with ſucceſs, I determined to make trial of it in ſmall doſes, and began by giving half a grain night and morning, with ten grains of cordial confection. This was continued for three days, when I increaſed the doſe to one grain night and morning, which ſhe continued for ten days without producing any effect either upon her mouth or bowels: her health ſhe thought ſomewhat better, at leaſt her ſpirits were ſo, when I added another grain to each of her boluſſes, which ſoon affected her mouth, ſo that I was under the neceſſity of diſcontinuing the medicine for ſome days. Her complaints, upon the whole, already wore a much more favourable aſpect: her ulcerated leg diſcharged but very little, and the ſwelling of both legs was leſſened. While the calomel was laid aſide, I made her take every evening a few tea-ſpoonfuls of *tinct. rhæi ſpirit*; but as ſoon as her mouth got well, ſhe again reſumed the uſe of the calomel, taking only two grains a day; in which way ſhe went on for ſeven weeks; her ſtrength gradually returning, together with her appetite; and all her complaints gradually diminiſhing. The calomel was now laid aſide, and, on the the ninth week from the taking of her boluſſes, ſhe had appearances of the menſtrual diſcharge. Riding on horſeback, together with the bark, were now recommended, and the following month ſhe menſtruated pretty freely, although not ſo much perhaps as ſhe had been accuſtomed to do when in health: the diſcharge, however, ſoon came to its uſual ſtandard; and I have only to add, that ſhe has been for four months pregnant.

